# Effect of Reiki therapy on quality of life among adolescents

**DOI:** 10.6026/973206300211375

**Published:** 2025-06-30

**Authors:** Gurav Priyankaben Rajeshbhai, Mahalakshmi B., Siva Subramanian N.

**Affiliations:** 1 Department of Pediatric Nursing, Nootan College of Nursing, Sankalchand Patel University, Visnagar, Gujarat - 384315; India; 2 Department of Psychiatric Nursing, Nootan College of Nursing, Sankalchand Patel University, Visnagar, Gujarat - 384315; India

**Keywords:** Reiki therapy, quality of life, adolescents, cancer

## Abstract

Adolescents with cancer face numerous challenges that impair their quality of life (QoL), including physical discomfort, emotional stress and disruptions to normal development. Therefore, it is of interest to study the impact of Reiki therapy on QoL among adolescents aged 13-17 years with cancer in Gujarat, India. A quasi-experimental pre-test and post-test control group design was employed with 92 participants, equally divided into experimental and control groups. Quality of life was assessed using the WHOQOL-100 tool. Post-intervention results demonstrated a significant improvement in the experimental group's QoL (Mean difference = 13.9, p < 0.001), while the control group showed minimal change. Thus, the potential of Reiki therapy as an effective intervention to enhance the QoL of adolescents undergoing cancer treatment is highlighted.

## Background:

Cancer during adolescence severely disrupts developmental milestones and diminishes quality of life (QoL) through physical discomfort, emotional distress and social withdrawal. Traditional cancer therapies like chemotherapy and radiation often exacerbate symptoms such as fatigue, pain and anxiety. To address these multidimensional challenges, complementary approaches like Reiki therapy have gained popularity in supportive oncology care [1]. Reiki is a Japanese energy healing practice that involves channelling energy via hand placements over the body to restore balance and promote healing. Evidence suggests that Reiki positively influences psychological and physical symptoms in cancer patients. A randomized trial among blood cancer patients demonstrated significant improvements in general, physical and environmental QoL domains [2]. Likewise, improvements in fatigue and anxiety were observed in breast cancer patients receiving Reiki alongside chemotherapy [3]. Systematic reviews and qualitative studies further support Reiki's therapeutic potential. Reiki has been linked to reduced pain and improved sleep in patients with advanced cancer [4], as well as emotional relief and increased life satisfaction [5]. Despite some methodological limitations, most findings suggest that Reiki is a safe, non-invasive therapy that enhances QoL outcomes, especially when used alongside conventional treatments [6, 7]. Given the unique vulnerabilities of adolescents, further studies are warranted to explore Reiki's impact in this demographic. Nonetheless, the current evidence base justifies its integration as a supportive therapy in pediatric oncology care [8]. Therefore, it is of interest to describe the effect of Reiki therapy on quality of life among adolescents.

## Methodology:

## Research design:

A quasi-experimental pre-test and post-test control group design was used to assess the effectiveness of Reiki therapy on quality of life among adolescents diagnosed with cancer.

## Research setting:

The study was conducted in selected cancer hospitals across Gujarat, India.

## Population and Sample:

The target population comprised adolescents aged 13-17 years who were undergoing cancer treatment. A total sample of 92 participants was selected using a convenience sampling technique, with 46 participants each in the experimental and control groups.

## Sampling criteria:

[1] Inclusion Criteria: Adolescents aged 13-17 years, diagnosed with cancer, willing to participate and capable of responding to the questionnaire.

[2] Exclusion Criteria: Participants with severe psychiatric illness, concurrent alternative therapy, or unwillingness to continue the sessions.

## Tools for data collection:

The WHOQOL-100 Questionnaire was used to measure the quality of life across multiple domains including physical, psychological, level of independence, social relationships, environment and spirituality/religion/personal beliefs.

## Intervention protocol:

The experimental group received Reiki therapy for 21 minutes daily over six consecutive days. The therapy was administered by a certified Reiki practitioner, focusing on energy balancing through the seven chakra points using standard hand placements. The control group received only routine care without any Reiki intervention.

## Data collection procedure:

Pre-test data were collected from both groups using the WHOQOL-100. Following the intervention period, post-test data were collected using the same tool. Participants were observed for compliance and comfort throughout the sessions.

## Data analysis:

Data were analyzed using SPSS version 25. Paired t-tests were conducted to compare pre- and post-intervention quality of life scores within each group, while independent t-tests were used to assess between-group differences. Additionally, chi-square tests were applied to evaluate associations between demographic variables and quality of life outcomes. A p-value < 0.05 was considered statistically significant.

## Results:

Table 1 (see PDF) shows that the majority of children in the experimental group were aged 13-15 years, male, had primary education, belonged to joint families and had fathers working in the private sector. Most mothers were housewives and monthly income was above ₹20,000. Retinoblastoma and Acute Lymphoblastic Leukemia were the most common cancers in this group. Table 2 (see PDF) shows that the majority of participants in the experimental group shifted from poor and moderate to high QoL after Reiki therapy, while the control group showed a decline in high QoL and an increase in poor and moderate levels post-test. Table 3 (see PDF) shows that the majority of improvement in mean QoL scores occurred in the experimental group after Reiki therapy, with a statistically significant difference (p < 0.001), whereas the control group showed minimal, non-significant improvement. Figure 1 (see PDF) shows that the majority of adolescents in the experimental group demonstrated a notable improvement in mean Quality of Life (QoL) scores after receiving Reiki therapy, increasing from 70.87 to 84.72. In contrast, the control group showed only a minimal increase in QoL scores, from 70.86 to 72.91. This highlights the statistically significant impact of Reiki therapy on enhancing the overall well-being of adolescents undergoing cancer treatment.

## Discussion:

The results of this study affirm that Reiki therapy significantly improves quality of life (QoL) among adolescents with cancer, supporting its role as a valuable complementary therapy. These findings align with a growing body of evidence indicating Reiki's efficacy in managing physical symptoms, emotional stress and treatment-related side effects across various cancer populations. Reiki therapy has been consistently associated with improvements in emotional well-being, reduced pain and alleviated fatigue. In a study by Orsak *et al.* (2014), cancer survivors reported reduced pain and depression along with significant QoL improvement following seven Reiki sessions [1]. Similarly, breast cancer patients receiving chemotherapy experienced significant reductions in fatigue and QoL enhancement after six Reiki sessions [3]. Reiki's effects on multidimensional well-being were confirmed in a randomized trial with blood cancer patients, which reported improved general, physical and environmental QoL domains [2]. Its role in managing cancer-related fatigue has been emphasized in multiple systematic reviews and trials, which found significant decreases in tiredness, pain and anxiety following Reiki sessions [9, 4]. Beyond the significant improvement in quality of life observed in this study, emerging research suggests that Reiki therapy may also benefit cognitive and emotional domains. A quasi-experimental study demonstrated that Reiki sessions led to statistically significant improvements in memory and behavioral symptoms in older adults with mild cognitive impairment and early Alzheimer's disease, using the AMMSE and RMBPC scales [10]. This opens new possibilities for Reiki's utility in supporting cognitive function and neuropsychological health. In the area of emotional intelligence and self-regulation, Reiki has been shown to improve positive emotions and reduce symptoms of anxiety, depression and agitation. A recent pilot study in a low-income mental health population revealed that even a single Reiki session significantly improved feelings of calmness, energy and happiness while decreasing stress and fatigue [11].

Moreover, a systematic review concluded that Reiki is more effective than placebo for managing clinical levels of depression, anxiety and stress, especially in those with diagnosable conditions [12]. Qualitative studies have deepened our understanding of Reiki's psychological benefits. Participants described emotional release, reduced anxiety and spiritual calm during sessions [5]. Such outcomes are particularly important in adolescents, who are highly sensitive to emotional disruption during illness. Further, Reiki has been found to enhance sleep quality-a critical component of QoL for patients undergoing hormone therapy and chemotherapy [13]. Similar benefits were seen in advanced cancer patients, with Reiki improving sleep and stress markers [4]. Systematic reviews reinforce these individual study findings. Guimarães *et al.* (2020) found improvements in fatigue, mood and pain across multiple cancers, highlighting Reiki's holistic impact [14]. Avcı and Gün (2023) similarly reported positive results in pain management across 572 cancer patients [15]. Other trials observed reduced analgesic need, improved relaxation and emotional uplift post-Reiki treatment, suggesting a decrease in the physiological stress burden [16, 17]. In the hospital setting, Reiki has also proven beneficial in reducing anxiety during chemotherapy sessions, as demonstrated in Orsak *et al.*'s trial on breast cancer patients [18]. REASSURE, a large-scale ongoing clinical trial in Germany, aims to validate these outcomes and investigate Reiki's effects on neuropathy during chemotherapy [19]. Reiki's integrative value in palliative and geriatric oncology settings cannot be overstated. It has facilitated improved comfort, calmness and dignity for terminally ill patients [20]. The study concludes that Reiki therapy is an effective, non-invasive intervention for improving the overall quality of life in adolescents undergoing cancer treatment. Participants who received Reiki showed significant improvements in physical, emotional and social well-being, as reflected in both categorical and mean QoL scores. A study by Díaz-Rodríguez *et al.* (2011) demonstrated that a single Reiki session significantly reduced salivary cortisol and improved mood states among healthy participants, suggesting its potential to modulate stress-response systems. Integrating Reiki in adolescent oncology settings could therefore help mitigate such hormonal imbalances, contributing to better resilience and adaptation to illness [21]. Jain and Mills (2010) conducted a systematic review comparing bio field therapies (including Reiki) across various clinical populations and found consistent improvements in pain perception, behavioral distress and general well-being. These findings are particularly significant for adolescents, who may be more emotionally vulnerable during invasive cancer treatments [22]. Liu *et al.* (2025) conducted a meta-analysis on Effects of Reiki therapy on quality of life and their result alogn with our study [23]. Reiki emerges as a safe and effective complementary therapy for adolescents with cancer, offering relief from pain, fatigue, anxiety and emotional distress. Its holistic approach supports both physical and psychological well-being, making it a valuable addition to supportive oncology care. Given its ease of use and minimal side effects, Reiki can enhance quality of life when integrated with conventional treatments. Further robust studies are needed to confirm long-term benefits and establish clinical guidelines.
